# The Effect of Metformin and Hydrochlorothiazide on Cytochrome P450 3A4 Metabolism of Ivermectin: Insights from In Silico Experimentation

**DOI:** 10.3390/ijms252212089

**Published:** 2024-11-11

**Authors:** Thuli R. Mtambo, Kgothatso E. Machaba, Nireshni Chellan, Pritika Ramharack, Christo J. F. Muller, Ndumiso N. Mhlongo, Nokulunga Hlengwa

**Affiliations:** 1 Department of Biochemistry and Microbiology, University of Zululand, Kwa-Dlangezwa 3886, South Africa; 2 Biomedical Research and Innovation Platform, South African Medical Research Council, Tygerberg 7505, South Africa; 3 School of Laboratory Medicine and Medical Sciences, University of KwaZulu-Natal, Durban 4001, South Africa; 4 Division of Medical Physiology, Faculty of Health Sciences, Stellenbosch University, Tygerberg 7505, South Africa

**Keywords:** SARS-CoV-2, COVID-19, diabetes, hypertension, drug–drug interactions, hepatic metabolism, ivermectin, metformin, hydrochlorothiazide, cytochrome P450 3A4

## Abstract

The spread of SARS-CoV-2 has led to an interest in using ivermectin (a potent antiparasitic agent) as an antiviral agent despite the lack of convincing in vivo clinical data for its use against COVID-19. The off-target prophylactic use of ivermectin adds a substantial risk of drug–drug interactions with pharmaceutical medications used to treat chronic conditions like diabetes and hypertension (metformin and hydrochlorothiazide, respectively). Therefore, this study aims to evaluate the potential drug–drug interactions between ivermectin with either metformin or hydrochlorothiazide. In silico experiments and high-throughput screening assays for CYP3A4 were conducted to understand how metformin and hydrochlorothiazide might affect CYP3A4’s role in metabolizing ivermectin. The study findings indicated that hydrochlorothiazide is more stable than both ivermectin and metformin. This conclusion was further supported by root mean square fluctuation analysis, which showed that hydrochlorothiazide is more flexible. The variation in the principal component analysis scatter plot across the first three normal modes suggests hydrochlorothiazide has a more mobile conformation than ivermectin and metformin. Additionally, a strong inhibition of CYP3A4 by hydrochlorothiazide was observed, suggesting that hydrochlorothiazide’s regulatory effects could significantly impede CYP3A4 activity, potentially leading to a reduced metabolism and clearance of ivermectin in the body. Concurrent administration of these drugs may result in drug–drug interactions and hinder the hepatic metabolism of ivermectin.

## 1. Introduction

The SARS-CoV-2 virus, the etiological agent of Coronavirus disease (COVID-19), has been considered to be one of the leading causes of death worldwide since 2019 [1]. The WHO estimated that 623 million people were living with COVID-19 and 6.56 million died of COVID-19 in October 2022 [2]. In addition, individuals with underlying conditions such as diabetes and hypertension were shown to experience more severe effects of COVID-19 [3].

The emergence of COVID-19 and its alarming infection and mortality rates have motivated the urgent need for new drugs. Hence, several drugs such as ivermectin have been examined for drug repurposing against COVID-19 [4]. Recent in vitro studies have demonstrated that ivermectin suppresses SARS-CoV-2 replication by inhibiting the importin (IMPα/β)–viral complex from entering the cell where viral replication occurs [5]. Hence, ivermectin became one of the potential drugs against COVID-19 with low effective dosage levels.

**Figure 1:**
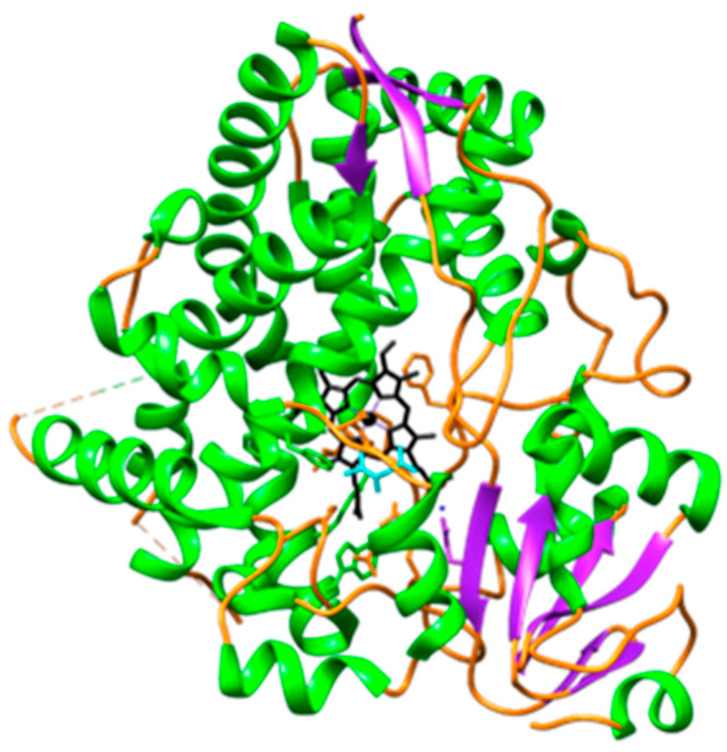
Illustration of the front view of CYP3A4 [11] in complex with the heme. The CYP3A4 chain is composed of helix (green), sheet (purple), and loop (orange) structures, with the heme depicted in black. The heme is positioned between the flexible loop regions of the protein, which are believed to play a crucial role in the mechanism of ligand dissociation and association [12].

Ivermectin, like most drugs, is primarily metabolized by cytochrome P450 enzymes. Cytochrome P450 enzymes (CYP450) are a superfamily of heme-containing enzymes involved in the phase 1 biotransformation of clinically relevant drugs [6]. These enzymes are not only involved in the metabolism of drugs but also in fatty acids, steroids, carcinogens, and xenobiotics [7]. An important characteristic of CYP450 enzymes is that they have a wide and overlapping substrate specificity [8] with different subfamilies. CYP3A4 (Figure 1) belongs to the CYP3 family which is the major enzyme predominantly found in the liver and small intestines and contributes to the first-pass metabolism of approximately 50% of drugs in clinical use and xenobiotics [9]. Hence, ivermectin is primarily metabolized by CYP3A4 into two major metabolites, namely, 3′-*O*-demethyl ivermectin and 4a-hydroxy ivermectin [10].

Amongst individuals who use ivermectin as an alternative solution for the treatment of COVID-19 are individuals with diabetes and hypertension. As a result, there are concerns about possible drug–drug interactions that may occur between ivermectin and other pharmaceutical drugs such as metformin and hydrochlorothiazide. Metformin is a biguanide derivative that is extensively used as a first-line treatment for type 2 diabetes [13]. It reduces hyperglycaemia through the inhibition of hepatic glucose production [14]. Hydrochlorothiazide is a thiazide-like diuretic, primarily used for its antihypertensive effect [15].

Drug–drug interactions (DDIs) occur when one drug modifies the disposition of the co-administered drug [16]. Hence, DDIs may either increase or reduce the efficacy of the drugs leading to toxic effects [17]. Studies have shown that the inhibition of metabolizing enzymes, particularly cytochrome P450s, is the major cause of harmful DDIs [18]. The use of the ivermectin as an alternative treatment for COVID-19 by individuals with diabetes and/or hypertension has motivated the necessity of evaluating the possible inhibition of CYP3A4. Hence, the binding of CYP3A4 to ivermectin, metformin, and hydrochlorothiazide prompted the need to perform a comprehensive analysis by applying a computational simulation approach.

Herein, we aim to provide insights into ivermectin, metformin, and hydrochlorothiazide’s binding to the CYP3A4 protein and molecular events taking place through the course of a simulation. To date, the conformational dynamics of drug–protein interaction have been extensively studied using computational simulations. To further validate our findings, the Vivid assay, which is a fluorescent-based assay, was conducted to assess the inhibition. The Vivid assay makes use of fluorescent P450 substrates that are efficiently metabolized by specific P450 isozymes, resulting in a product with altered fluorescent properties, and typically an increased fluorescent intensity [19].

To the best of our knowledge, this is the first account of such a study on ivermectin, metformin, and hydrochlorothiazide’s binding to the CYP3A4 protein. Hence, we believe the findings reported in this study may improve our understanding of the CYP3A4 protein binding landscape, which in turn could pave the way for an understanding of the use of ivermectin as an alternative treatment for COVID-19 by individuals with diabetes and/or hypertension.

## 2. Results and Discussions

**Figure 2:**
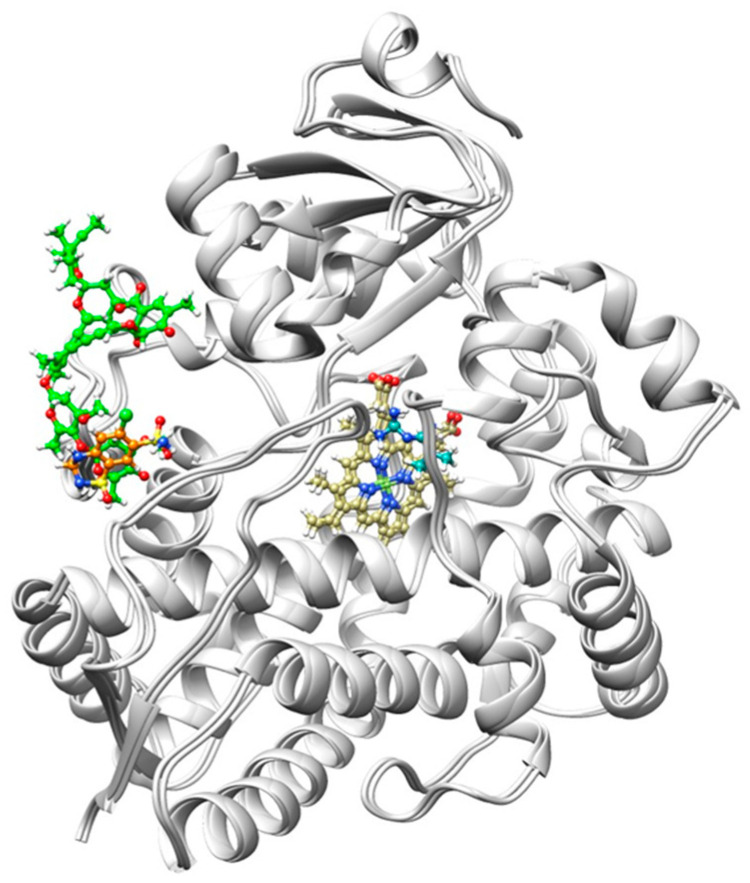
Illustration of ivermectin, metformin, and hydrochlorothiazide in terms of their binding orientation. Metformin (Cyan) displays a known binding site located at the center of the CYP3A4 protein (light gray) next to the Heme (dark khaki). On the other hand, ivermectin (green) and hydrochlorothiazide (orange) display unknown binding sites. Hence, the results suggest that the CYP3A4 protein has more than one binding site.

Molecular docking, a computational method employed to predict binding affinity by analyzing the interactions between a protein and small molecules [20]. Recently, docking has been applied to identify the binding site of a biological target [21]. In the current work, molecular docking was carried out on ivermectin, hydrochlorothiazide, and metformin with CYP3A4 and the results are presented in Table 1 and Figure 2. Hence, Lipinski’s rule of five was taken into consideration.

The results (Table 1) presented in the current study reveal that all of the docked drugs ranged from −4.4 to −9.3 kcal/mol. Ivermectin showed the highest DS of −9.3 kcal/mol, while hydrochlorothiazide and metformin displayed lower DSs of −5.9 to −4.4 kcal/mol, respectively. It was also notable that the cLogP of ivermectin was higher than the normal range which is between −2 to 3. The cLogP is the lipophilicity of the compound that usually influences its permeability, hepatic clearance, or solubility. cLogP values ranging from −2 to 3 exhibit the highly favorable potential of achieving permeability and first-pass clearance. According to the literature, compounds with a higher molecular weight usually have higher cLogP values that are greater than 4. And, therefore, we can conclude that the favorable contributions towards the DSs of all drugs were from MW and cLogP. Hence, our results suggest that CYP3A4 strongly binds to non-polar drugs with high MWs.

**Figure 3:**
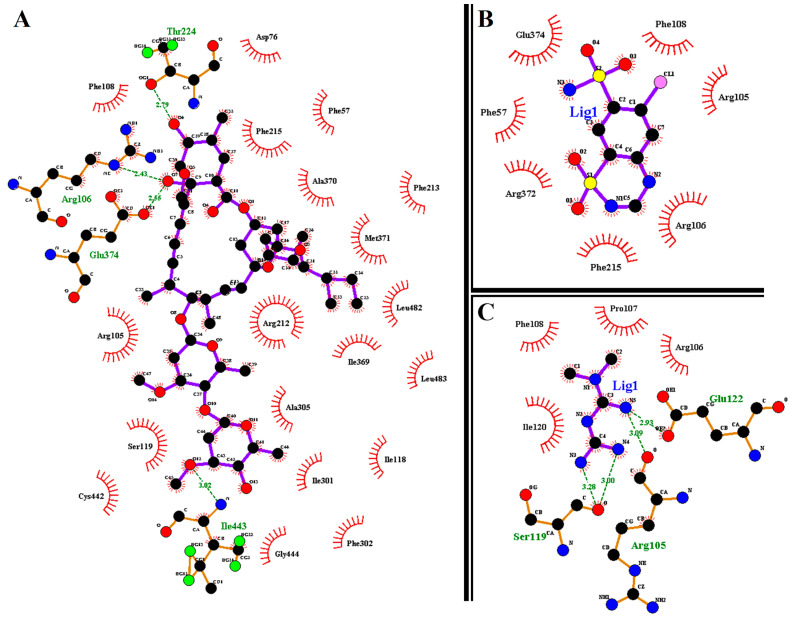
Ligand–residue interactions of (**A**) CYP3A4-IVM, (**B**) CYP3A4-MET, and (**C**) CYP3A4-HCTZ complexes. The red eyelashes represent the hydrophobic interactions; green dotted lines (H-bonds); black (carbon); red (oxygen); and blue (nitrogen).

We provide further insight into the individual amino acids’ contributions to the binding of (IVM), hydrochlorothiazide (HCTZ), and metformin (MET); the results are presented in Figure 3. Briefly, this allowed us to characterize the behavior of small molecules in the protein’s binding site and elucidate fundamental biochemical processes [22].

The results obtained from ligand–residue interactions show that the oxygen atoms in IVM create hydrogen bond interactions with ILe443, Glu374, Arg106, and Thr224 while the nitrogen atoms in HCTZ create hydrogen bond interactions with Arg105, Ser119, and Glu122. On the other hand, MET displayed no hydrogen bond interactions. IVM also shows nineteen hydrophobic interactions with CYP3A4 active site residues while HCTZ and MET displayed seven and four hydrophobic interactions, respectively. In addition, this work supports the research that suggested that more rings in a structure enhance hydrophobicity on a molecular level [23]. Based on the information presented in Figure 3 and Table 1 for the IVM, MET and HCTZ complexes, we can conclude that the hydrophobic interaction of IVM with CYP3A4 active site residues leads to higher binding energy of −9.3 kcal/mol [24]. Hence, the findings reported in this study enhance our understanding of IVM, MET, and HCTZ bound to CYP3A4.

### 2.1. Stability and Flexibility of the Systems

In the current study, we carried out root mean square deviation (RMSD), root mean square fluctuation (RMSF), and radius of gyration (RoG) measurements to gain insight into the stability and flexibility of the CYP3A4 protein in complex with IVM, MET, and HCTZ. The RMSD calculation was carried out during a 50 ns simulation, to ensure that all systems were well equilibrated before further post-MD analysis. RMSF provides insight into the flexibility of the protein structure regions while RoG gives insight into the compactness of protein structures during the MD simulation [25].

**Figure 4:**
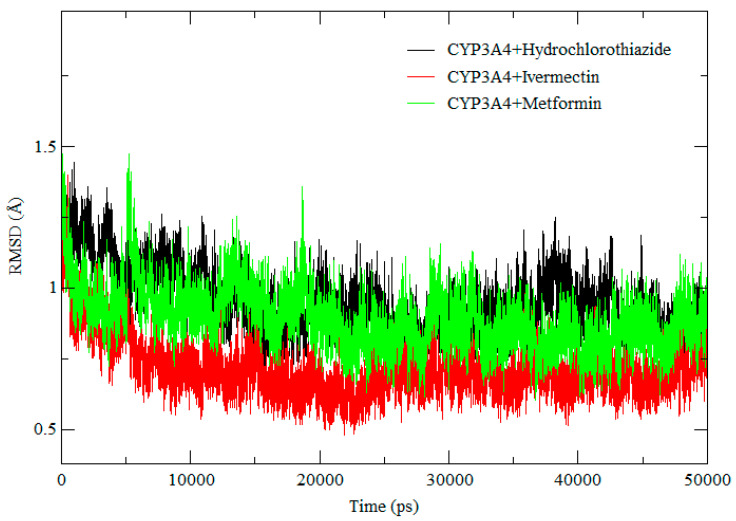
RMSD of CYP3A4-HCTZ, CYP3A4-IVM, and CYP3A4-MET.

The RMSD plots of IVM, MET, and HCTZ in complex with CYP3A4, as illustrated in Figure 4, show that IVM achieved a lower RMSD value compared to MET and HCTZ throughout the simulation. Hence, it was observed that all systems were well stabilized throughout the simulation which confirms the validity of the results. In addition, changes in the RMSD flexibility of the CYP3A4, suggest that the presence of IVM, MET, and HCTZ affect the function of the CYP3A4. To gain a more specific insight into the protein structural changes, we applied RMSF to determine the amino acid flexibility.

**Figure 5:**
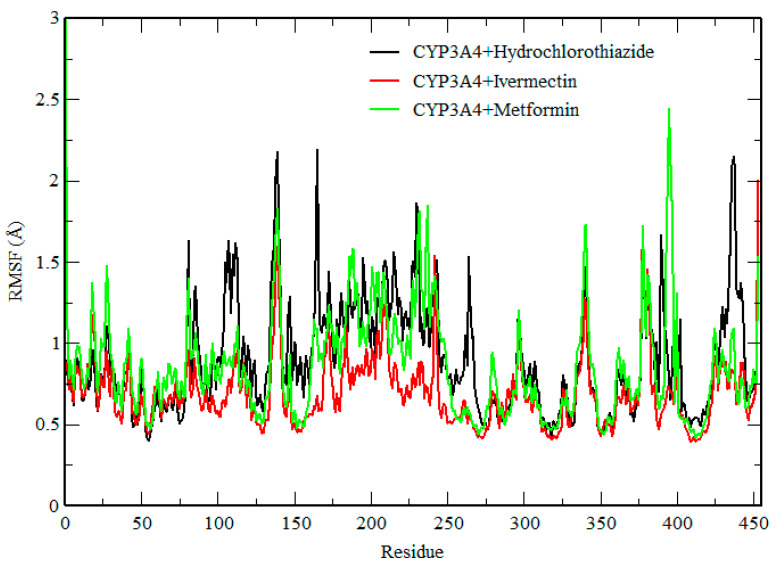
RMSF of CYP3A4–hydrochlorothiazide, CYP3A4–ivermectin, and CYP3A4–metformin.

RMSF measures the fluctuations in each carbon atom in the protein and it offers insight into their flexibility [26]. Here, we assessed the dynamic behavior of each amino acid (Figure 5) within the protein structure [27]. The complexes showed a similar trend of conformational flexibility. However, the presence of IVM in the active site of the protein reduced the flexibility of the amino acids compared with metformin and hydrochlorothiazide. In the case of CYP3A4-HCTZ, higher fluctuations were seen in the following regions, THR139, ASN165, and LEU438, while in the case of CYP3A4-IVM, higher fluctuations were observed in the THR139 and ASP453 regions. In the case of CY3A4-MET, fluctuations were observed in the following regions: HIE1, THR139, and PRO395. These results suggest that CYP3A4 is highly flexible during the metabolism of IVM and, therefore, in conclusion, the fluctuations observed in this system revealed that these drugs interfere with the activity of CYP3A4.

**Figure 6:**
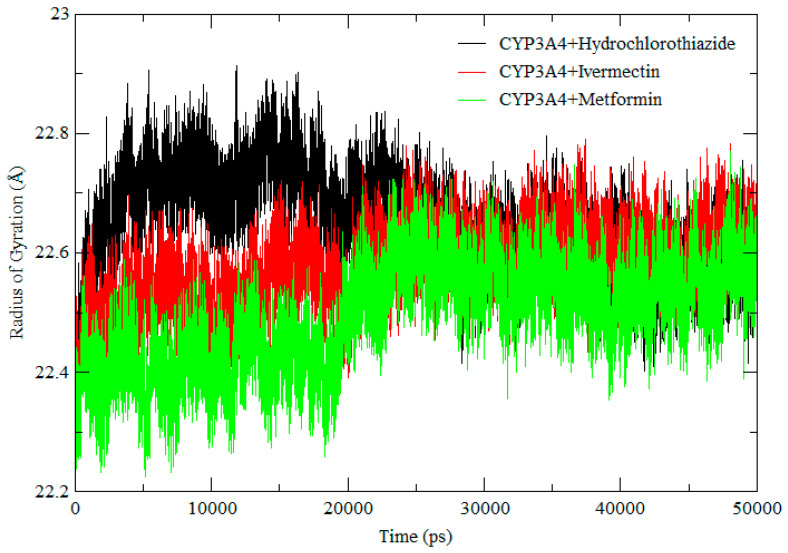
RoG comparison across the 500 ns molecular dynamic simulation of CYP3A4 + hydrochlorothiazide (black), CYP3A4 + ivermectin (red), and CYP3A4 + metformin (green).

Figure 6 shows the conformational stability of the protein structures and compactness during the simulation. Throughout the simulation, all complexes showed a very similar Rog, meaning that the system was stable throughout the simulations. These results are well correlated to those of the RMSD and RMSF, which suggested a similar trend in molecular flexibility and a minimal difference in residual mobility of the protein structures in the respective systems. Therefore, based on these results we can conclude that the results are reliable, and the compounds are less likely to interfere with the activity of CYP3A4 as the system was stable throughout.

### 2.2. Screening of Cytochrome P450 Enzyme Inhibition

**Figure 7:**
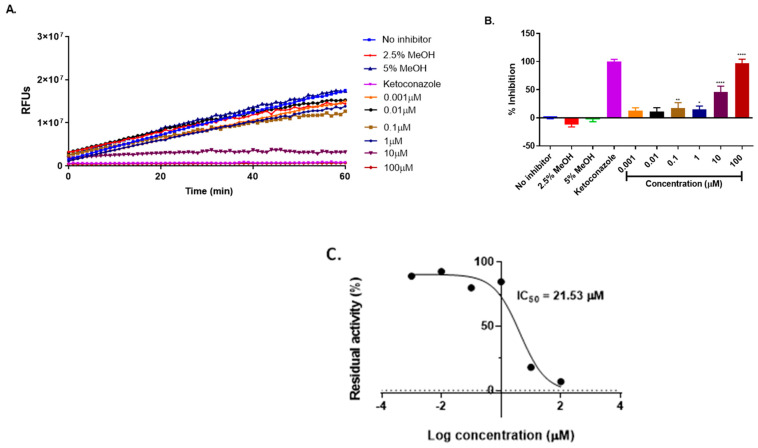
Inhibition of BOMC transformation by CYP3A4 in the presence of ivermectin (**A**). The rate of enzyme inhibition over 60 min of incubation (**B**) and the remaining enzyme activity (**C**). The results are from three independent experiments conducted in triplicates (*n* = 3) and are expressed as mean ± SD. One-way ANOVA was used for the statistical analysis, where **** = *p* <0.0001, ** = *p*< 0.006, and * = *p* = 0.05, respectively.

**Figure 8:**
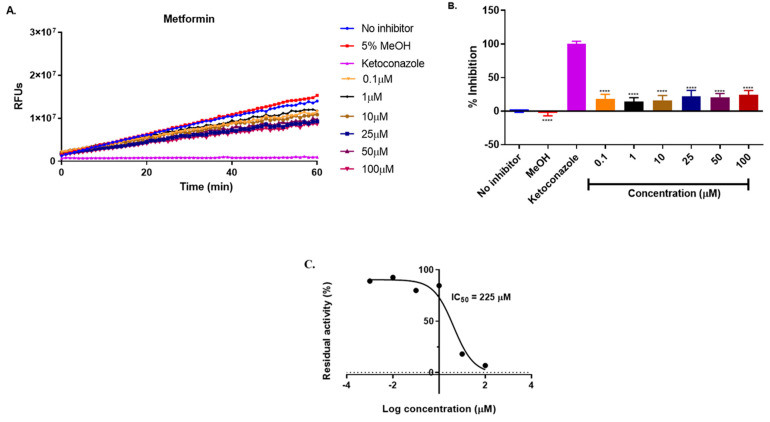
Inhibition of BOMC transformation by CYP3A4 in the presence of metformin (**A**). The rate of enzyme inhibition over this incubation period is shown in (**B**). The residual enzyme activity (**C**). The results are based on three independent experiments conducted in triplicate (*n* = 6) and are presented as mean ± SD. Statistical analysis was performed using one-way ANOVA, with significance levels indicated as **** = *p* < 0.0001.

**Figure 9:**
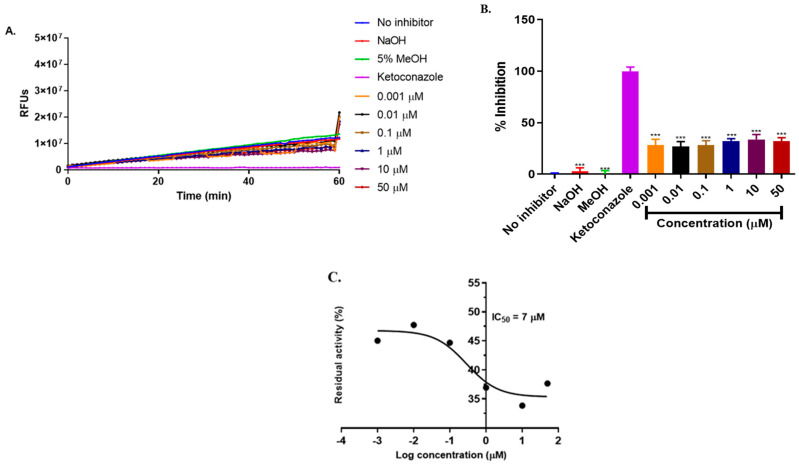
Inhibition of BOMC transformation by CYP3A4 in the presence of hydrochlorothiazide (**A**) The rate of CYP3A4 inhibition over 60-minute incubation (**B**) and the remaining enzyme activity (**C**). The results are presented as the mean SD of three independent tests performed in duplicates (*n* = 3), and CYP inhibition is shown as a percentage of the vehicle control with a value of 100 per cent. One-way ANOVA was used for the statistical analysis, where *** *p* < 0.001.

CYP3A4 is the major enzyme responsible for the metabolism of ivermectin. The inhibition of CYP3A4 by ivermectin, metformin, and hydrochlorothiazide was analyzed, with the results presented in Figure 7, Figure 8 and Figure 9.

According to [28], a compound with an IC_50_ value less than 10µM is considered a strong inhibitor, whereas a compound with IC_50_ value between 10 and 50µM is a moderate inhibitor and IC_50_ greater than 50µM is a weak inhibitor. Ivermectin is moderately metabolized by CYP3A4 in the liver [29]. Our study revealed that ivermectin exhibited a moderate inhibition of CYP3A4 with IC_50_ = 21.53µM (*p* < 0.0001) (Figure 7) compared to ketoconazole which is a known inhibitor of CYP3A4 which showed 100% inhibition of CYP3A4. However, in combination with metformin and hydrochlorothiazide, a strong induction was observed. This interaction may not affect the metabolism of ivermectin but may contribute to drug–drug interactions. Metformin alone (Figure 8) displayed a weak inhibition of CYP3A4 with IC_50_ = 225 μM, indicating that there are minimal chances for metformin to interfere with the hepatic metabolism of ivermectin when concurrently administered. On the other hand, hydrochlorothiazide (Figure 8) showed a strong inhibition of CYP3A4 with an IC_50_ value of 7µM (*p* < 0.0001). Hence, our results suggest that hydrochlorothiazide may interfere with the hepatic metabolism of ivermectin when administered concurrently.

## 3. Materials and Methods

### 3.1. Preparation of System for Molecular Docking

The X-ray crystal structure of CYP3A4 was obtained from Protein Data Bank (PDB) [30] with PDB code 5G5J [11]. All non-standard residues were removed or deleted from the protein during protein preparation in Chimera [31]. Ivermectin (ID: 45114068), metformin (ID: 4091), and hydrochlorothiazide (ID: 3639) 3D structures were obtained from PubChem {https://pubchem.ncbi.nlm.nih.gov (accessed on 12 October 2021)} [32] and optimized in Avogadro software {https://avogadro.cc (accessed on 21 July 2021)} [33].

### 3.2. Molecular Docking

Molecular docking of ivermectin, metformin, and hydrochlorothiazide into CYP3A4 was performed using the Autodock vina tool [34]. Blind docking was performed and the grid box was analyzed with the following grid parameters, x = 72 Å, y = 110 Å, and z = 98 Å, for the dimensions, while for the center grid was x = 20.58 Å, y = −24.83 Å, and z = 11.86 Å and with exhaustiveness = 8 which covered the entire region occupied by the ligands at the active site of the protein. UCSF Chimera was used for the visualization of all docked complexes [31]. In this study, all docked complexes were chosen to be subjected to the molecular dynamic simulation simulations.

### 3.3. Molecular Dynamic Simulations

Molecular dynamic simulations were conducted using the Amber14 software package’s GPU version of the PMEMD engine, as previously described in our previous work [25]. The atomic partial charges for the compounds were generated using the Antechamber module, while the LEAP module was used to add counter ions and hydrogen atoms to the protein. The system was contained in a TIP3P water box, with 10 Å between the system surface and the box boundary. The system was initially minimized for 2500 steps before being gradually heated from 0 to 300 K with 1ps and 5 kcal mol1 2 (collision frequency and harmonic restraints, respectively) settings using a Langevin thermostat. At 300 K and a 1 bar constant pressure, the system was equilibrated with no restrictions, and the SHAKE algorithm restricted the system’s bonds with hydrogen atoms. A 50 ns MD was performed in an isothermal-isobaric ensemble using a Berendsen barostat at 1 bar pressure and a pressure-coupling constant of 2 ps. The same procedure was followed for all other systems.

The MD trajectories were then subjected to post-analysis calculations after the 150 ns simulations were completed using the Amber14 modules PTRAJ and CPPTRAJ. MM-PBSA (molecular mechanics Poisson–Boltzmann surface area), ligand–residue interactions, hydrogen bonding, RMSD (root mean square deviation), RMSF (root mean square fluctuation), and Rog (radius of gyration) were analyzed.

### 3.4. Screening of Recombinant Cytochrome P450 Activity

The inhibitory effect of ivermectin, metformin, and hydrochlorothiazide on cytochrome P450 3A4 enzymes was assessed using Vivid^®^ screening kits (blue) (Catalog P. Life Technologies Corporation: Carlsbad, CA, USA, 2012). The experiments were conducted using the manufacturer’s protocol [35]. For each assay, two master pre-mixes were used, Master Pre-Mix I and II. Master Pre-Mix I contained Vivid^®^ reaction buffer I (200 mM potassium phosphate), BACULOSOMES ^®^ Plus reagent, and the Vivid^®^ regeneration system (333 mM glucose-6-phosphate. pH 8) while Mater pre-mix II contained the Vivid^®^ Reaction buffer I, the Vivid^®^ blue reconstituted substrate (7-benzyloxymethoxy-3-cyano-coumarin (BOMCC), and the Vivid^®^ NADP^+^. Ketoconazole was used as a positive inhibitor, as suggested by the manufacturer. Test compounds and a positive inhibitor were dissolved in different solvents (ivermectin: 2.5% MeOH, hydrochlorothiazide: 0.00014 M NaOH, metformin: water) and ketoconazole was dissolved in 0.1% MeOH. Assays were conducted in a 96-black-walled, clear-bottom plate in endpoint and kinetic mode. All assays were performed in duplicate, and the reaction was stopped by adding 50 μL tris base, followed by fluorescence measurement using the SpectraMax i3x (Molecular Devices, LLC, Sunnyvale, CA, USA) at 415 nm excitation and 460 nm emission wavelengths.

### 3.5. Time-Dependent Screening of the Compounds

Serial dilutions of ivermectin (concentration range 100–0.001 μM), metformin (100–0.1 μM), and hydrochlorothiazide (50–0.001 μM) were added to CYP3A4 to determine their IC_50_. Master pre-mix 1 (as described above) was added to a 96-black-walled, clear-bottom plate containing the test compounds. The plate was incubated at 37 °C for 15 min. After 15 min, the reaction was then initiated by the addition of Master Pre-Mix II containing the CYP3A4 reconstituted substrate, NADP^+^, and reaction buffer; following the incubation at 37 °C for 60 min, the reaction was terminated by the addition of 50 μL of tris base and then fluorescence was measured using the appropriate excitation and emission wavelengths.

### 3.6. Screening of Cytochrome P450 Enzyme Inhibition

The data generated were exported to an Excel worksheet. In the kinetic experiments, the reaction rate was determined by examining the variation in fluorescence over time. The following formula was used to calculate the percentage inhibition of the test compounds, positive inhibitor, and solvents.

$$\left(1-\left(\frac{Test\;compound-positive\;inhibitor}{Solvent\;control-positive\;inhibitor}\right)\right)\times 100\%$$

The remaining enzyme activity was calculated using the following formula:

$$Residual\;activity=\left(\frac{Test\;compound}{positive\;control}\right)\times 100\%$$

### 3.7. IC_50_ Determination

GraphPad Prism^®^ version 6 (GraphPad Software Inc., La Jolla, CA, USA) software was used to calculate the IC_50_ values. In a line graph, the percentage inhibition of the enzyme was briefly displayed versus the chemical concentration. To analyze the plot, non-linear regression was used (curve fit). Following the selection of dose–response inhibition, the plot’s variable slope (composed of three factors) was analyzed.

### 3.8. Statistical Analysis

The results are presented as the mean ± SD of three independent experiments performed in duplicate (*n* = 8), and CYP inhibition is shown as a percentage of the vehicle control. One-way ANOVA was used for the statistical analysis, Where *** *p* <0.001, ** *p* <0.01, and * *p* < 0.05, respectively. Where *p* < 0.05 is considered significant.

## 4. Conclusions

In this study, we have demonstrated that CYP3A4 has a strong binding affinity for non-polar drugs with a high molecular weight and that the presence of ivermectin (IVM), metformin (MET), and hydrochlorothiazide (HCTZ) influences CYP3A4 function, as indicated by inhibition observed in the vivid assay. The combination of ivermectin with hydrochlorothiazide and metformin presents a significant potential for interaction. The concurrent use of these drugs may lead to drug–drug interactions and hinder the hepatic metabolism of ivermectin. However, further research is needed to fully understand the mechanisms underlying these interactions.

## Figures and Tables

**Table 1:** Details of the docking studies for ivermectin, hydrochlorothiazide, and metformin.

Drug Name	2D Structure	DS	cLogP	HBD	HBA	MW (g/mol)	RB
Ivermectin	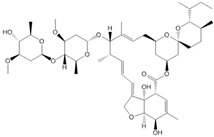	−9.3	4.4	3	14	875.1	8
Hydrochlorothiazide	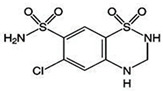	−5.9	−0.2	3	6	297.741	1
Metformin	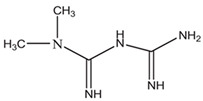	−4.4	−0.9	3	2	129.164	2

cLogP (consensus LogP), HBD (Hydrogen Bond Donor), HBA (Hydrogen Bond Acceptor), MW (molecular weight), DS (docking score: kcal/mol), and RB (Rotatable Bonds).

## Data Availability

The original contributions presented in the study are included in the article, further inquiries can be directed to the corresponding author/s.
